# Novel Application of Confocal Laser Scanning Microscopy and 3D Volume Rendering toward Improving the Resolution of the Fossil Record of Charcoal

**DOI:** 10.1371/journal.pone.0072265

**Published:** 2013-08-15

**Authors:** Claire M. Belcher, Surangi W. Punyasena, Mayandi Sivaguru

**Affiliations:** 1 Department of Geography, University of Exeter, Exeter, United Kingdom; 2 Department of Plant Biology, University of Illinois, Urbana, Illinois, United States of America; 3 Institute for Genomic Biology, University of Illinois, Urbana, Illinois, United States of America; University of Zurich, Switzerland

## Abstract

Variations in the abundance of fossil charcoals between rocks and sediments are assumed to reflect changes in fire activity in Earth’s past. These variations in fire activity are often considered to be in response to environmental, ecological or climatic changes. The role that fire plays in feedbacks to such changes is becoming increasingly important to understand and highlights the need to create robust estimates of variations in fossil charcoal abundance. The majority of charcoal based fire reconstructions quantify the abundance of charcoal particles and do not consider the changes in the morphology of the individual particles that may have occurred due to fragmentation as part of their transport history. We have developed a novel application of confocal laser scanning microscopy coupled to image processing that enables the 3-dimensional reconstruction of individual charcoal particles. This method is able to measure the volume of both microfossil and mesofossil charcoal particles and allows the abundance of charcoal in a sample to be expressed as total volume of charcoal. The method further measures particle surface area and shape allowing both relationships between different size and shape metrics to be analysed and full consideration of variations in particle size and size sorting between different samples to be studied. We believe application of this new imaging approach could allow significant improvement in our ability to estimate variations in past fire activity using fossil charcoals.

## Introduction

Wildfires form unique products that interact with the carbon and nutrient balance of our planet. Some of these products (e.g. chars, soots and chemical signatures) are traceable in soils, sediments and ancient rocks and provide us with a record of Earth’s past fire history. Of these products, fossil charcoal in sediments and rocks provides the most unequivocal evidence of past wildfire events [1], [2]. Charcoals can either be washed and sieved out of sediments or released from rock samples via acid digestion (e.g. [2], [3]). In both cases, samples are sieved (at either 125 µm, 150 µm or 180 µm) to split the residues into microfossil and mesofossil fractions. Typically, the microfossil fraction is mounted into a palynological slide and studied using a transmitted light microscope and the mesofossil fraction studied using a low-power stereo microscope [2], [3]. The number of charcoal particles are then counted by a researcher working at the microscope and/or from 2-dimensional images captured digitally.

When digital images are used, the number and the surface area of the charcoal particles can be quantified using an image analysis system. Such systems rely on the contrast between the dark, opaque, essentially black colour of charcoal particles to distinguish them from other organic material in the sample. This allows lighter particles to be screened out using a simple pixel intensity threshold. A popular image analysis program often used for this purpose is Image J (http://rsbweb.nih.gov/ij/). This technique works best for charcoals picked from Pre-Quaternary mesofossil fractions or with Quaternary-aged samples where there are no coalified particles, as coal also appears black in digital images and cannot be distinguished from charcoal by image analysis software. Such methods allow the abundance of charcoal particles and/or the area of charcoal per volume of sediment or per gram of rock to be estimated in different samples throughout historical and geological time. These variations in charcoal abundance are taken to represent variations in fire activity often in response to environmental or ecological changes (e.g. [4], [5]). Good reviews of the methods typically used to quantify charcoal abundances in peats, sediments and rocks can be found in [2], [6], [7], [8], [9], [10].

It is well known that peaks in mesofossil charcoal abundances are able to reflect incidences of wildfires within a watershed in recent sediments (e.g. [8]). However, the further studies go back in time, the more difficult it becomes to assess the relationship between an individual fire event and the record of fossil charcoals. Part of the problem in interpreting such records is due to differential fragmentation of charcoal particles during the processes of transport and deposition. For example, consider two wildfires with exactly the same properties, the same vegetation, the same burn conditions (e.g. climate, weather, fire temperature, spread rate etc.) and which create the same number of particles of charcoal. Following the fire event the charcoal particles are transported from the site via different means but deposited in the same sedimentary environment. In the first example, the charcoals are washed gently via overland flow into a lake whereas in the second example the charcoal is washed into a river, transported as bedload and later deposited in the lake. The first example might cause relatively little fragmentation of the original charcoal particles, whereas the second option would most likely cause significant fragmentation. This would thereby apparently increase the number of charcoal particles deposited. Although the charcoal would be considered to occur in isotaphonomic sedimentary units (i.e. lacustrine sediments), the means by which they were deposited into the lake would strongly have influenced the fragmentation of the charcoal prior to deposition. This could lead to the sample containing the greater number of charcoal particles per volume of sediment as being interpreted as a more significant peak in fire activity.

Measuring the total surface area of the charcoal particles in such samples goes some way to addressing the problem of fragmentation, as is often attempted with Quaternary charcoal material [10]. However, the use of total area still potentially misses the 3-dimensional aspect of the particles such that fragmentation into thin versus thick particles could entirely miss the true variation in total charcoal volume.

Here we use a novel application of confocal laser scanning microscopy and image processing to reconstruct the volume of individual charcoal particles such that they can be expressed as total volume of charcoal in a given volume of sediment or per gram of rock. Our method images charred particles in both microfossil and mesofossil slides using reflected laser light and captures 3-dimensional images of individual particles within slides. Using image-processing techniques, we are able to reconstruct the volume of each particle within a slide. We also quantify particle area, compare it to particle volume and consider the variety of morphological variation in particles in a given sample, in order to highlight possibilities for volume reconstructions using standard, readily available light microscope techniques. We believe application of this new imaging approach and what we can learn from the relationships that it reveals, may allow significant improvement in our ability to provide quantitative estimates of fossil charcoal abundance by enabling researchers to account for possible differential fragmentation of particles between samples.

## Materials and Methods

### Ethics Statement

No permits were required for the described study, which complied with all relevant regulations. The Cretaceous aged charcoal particles used in this analysis were collected from the Potomac Group rocks of the Rocky Point locality in Maryland by Claire Belcher (The University of Exeter). The modern Miombo woodland charcoal was provided by Casey Ryan (The University of Edinburgh). The large pieces of modern charcoal were provided by Margaret Collinson (Royal Holloway University of London) and were created in a bonfire from foliage clippings from her garden. None of the material used is listed as endangered or threatened by the IUCN.

### Samples and Sample Preparation

Three different types of charcoal sample were prepared in order to capture a range of charred materials and the typical range of methods used to quantify charcoal particles from Quaternary sediments and Pre-Quaternary rocks. The samples were: 1) large fragments of angiosperm wood charcoal made in a bonfire, 2) small fragments of *Pterracarpus angolensis* charcoal collected from a modern wildfire that occurred in a Miombo woodland in Africa (see [11]) and 3) ancient macrofossil charcoal fragments of Cretaceous age of unknown plant origin from the Rocky Point locality in Maryland, USA. These samples provided both modern and fossil examples of both microfossil and mesofossil size fractions with the aim being to highlight application of the methodology to both the modern, Quaternary and Pre-Quaternary sciences. All samples (both micro- and mesofossil fractions) were mounted onto slides (as is required for confocal laser scanning microscopy) with the particles dispersed in silicone oil [a mounting media typically used in the Quaternary sciences [12]], and covered with a 1.7 cm wide by 1.7 cm long cover slip and sealed with clear nail polish.

Three smaller fragments of the bonfire charcoal were chipped off the original samples and mounted into three slides, the purpose being to make relatively large pieces that could be measured in length, width and depth manually and to test the volume estimates provided by the machine. The Miombo woodland wildfire sample was sieved through a 150 µm sieve and the fine and large fractions collected. The fine fraction serves to represent the typical size fraction of charcoal as quantified in palynology slides and the larger fraction a typical modern mesofossil fraction. Both fractions were mounted into several slides of even dispersion. Slides of even dispersion were made by dispersing the charcoal residues in 6 ml of water and mounting 100 µl of the charcoal-water solution onto a cover slip. The aliquot was dried slowly on a hot plate at low heat to make sure particles adhered in an even fashion over the surface of the cover slip. The cover slip was then attached to a slide using silicon oil and sealed with nail polish.

The Cretaceous charcoal particles were released from a 5 gram sample of rock via standard processing techniques. The rock was demineralised by processing for 24 hours in cold 37% hydrochloric acid to remove any carbonates and then for 72 hours in cold 40% hydrofluoric acid to remove silicates and returned to cold 37% hydrochloric acid to remove any potential calcium fluoride precipitates. The sample was rinsed and neutralised and passed through a 150 µm sieve and the large fraction collected. All the particles released were dispersed in a petri dish in a very small amount of water and the charred particles picked and mounted into slides of even dispersion following the method mentioned above. All the charred particles from the entire 5 gram sample were mounted onto slides with the same volume of residue dispersed on to each so that a representative amount of charcoal was represented in each slide. This sample was used to represent a typical ancient macrofossil charcoal sample as would be released from a small amount of rock.

### Manual Estimates of the Volume of Large Bonfire Fragments

The individual bonfire fragments of charcoal prepared in three separate slides were manually estimated during set up of the confocal scanning (see below). This allowed the particles length and width to be measured from the images and the depth by focusing through axial focal planes. The length, width and depth were multiplied together to give a very crude manual estimate of the volume of the particles and the length and width to provide area measurements.

### Confocal Laser Scanning Microscopy and 3D Volume Rendering of Charcoal

**Figure 1:**
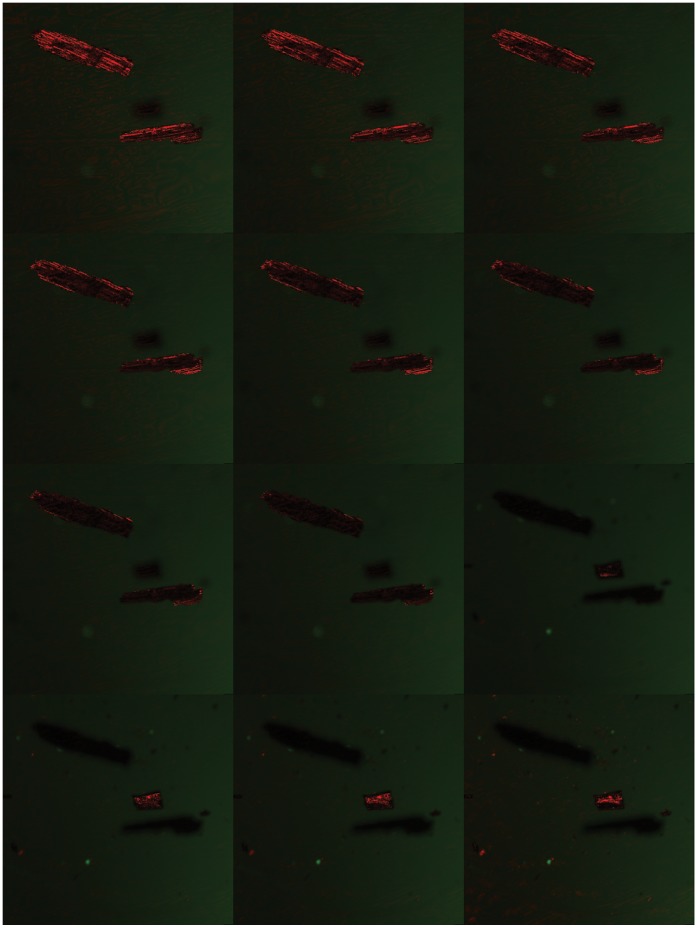
Series of images taken through different focal plains of 2 particles in reflected light.

**Figure 2:**
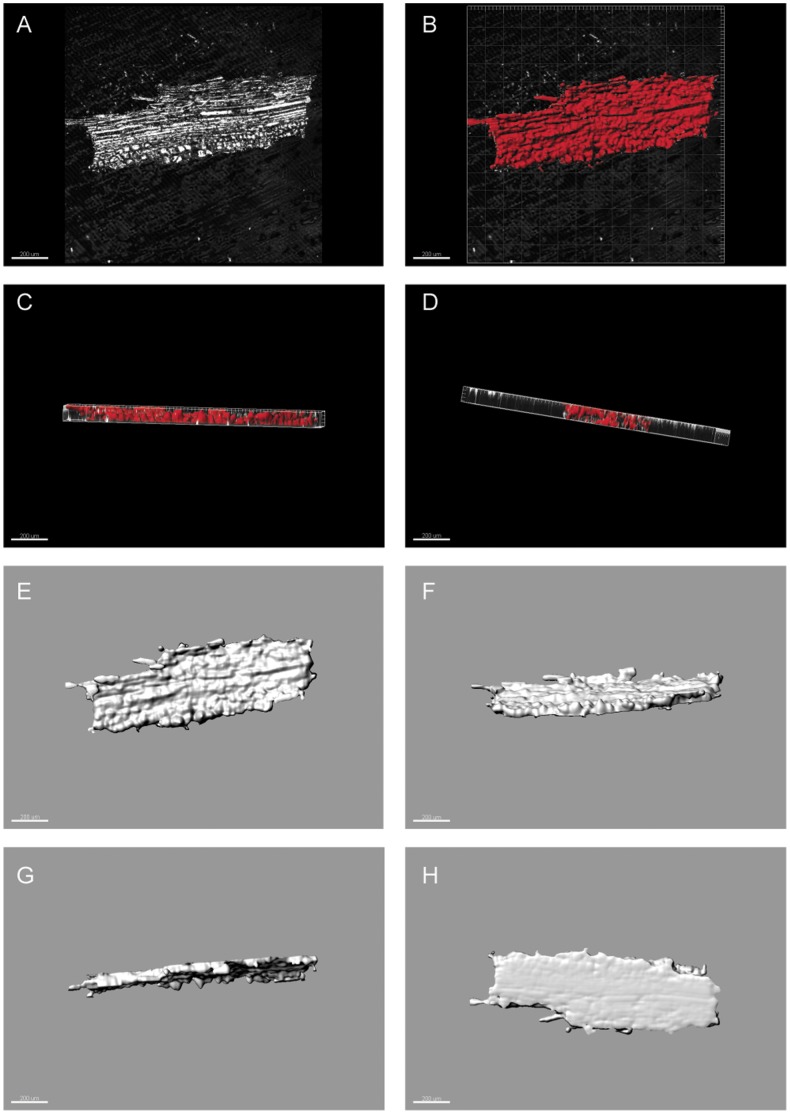
Three dimensional recontruction of Large “bonfire” particles of charcoal. (A) Original reflected light image (B–D) image processed three-dimensional reconstructions top view (X–Y) and side views (Z) (E–H) all views of the particle. Scale bars all 200 µm. In image H the particle appears to have a flattened base this flattening of the very large mm scale bonfire charcoal, which would not typically be imaged in a slide, is due to reflected light bouncing back from the slide of the cover slip. As such the final layer of this image has been deleted in the 3D images so that the particles stand out from the background. This is not a problem in the size range of particles normally mounted in slides (e.g. meso and micro fossil fractions).

**Figure 3:**
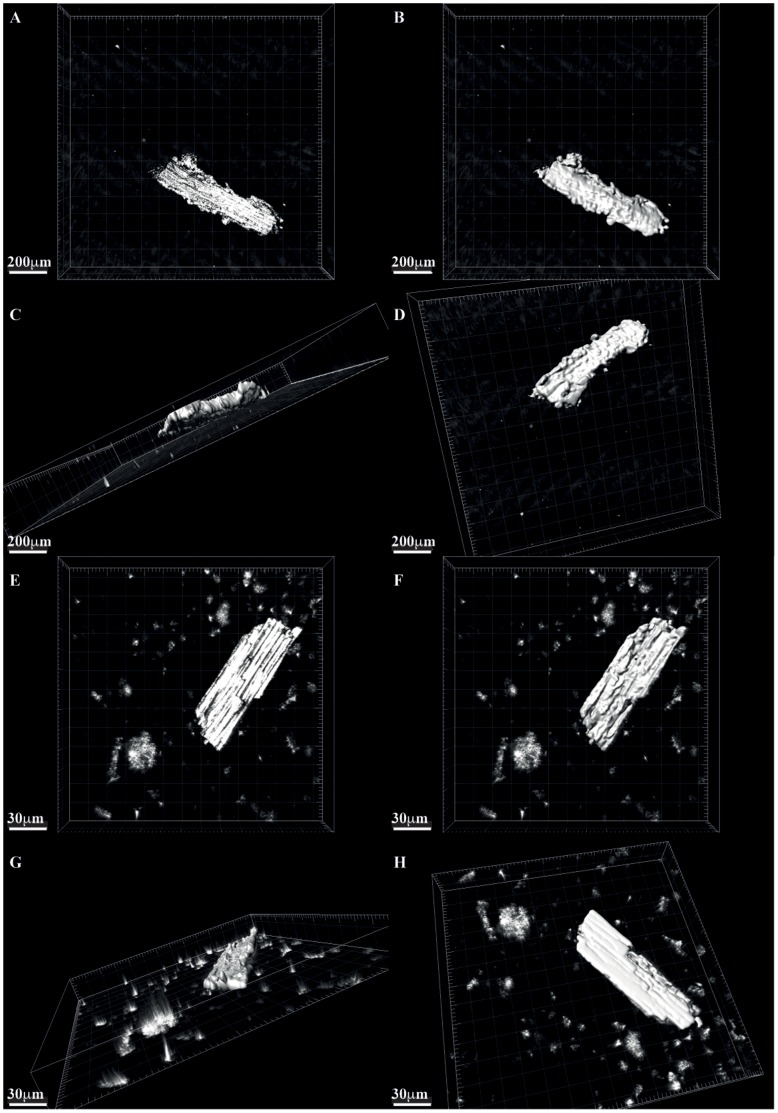
Images showing reconstructions of charcoals. (A–D) Example modern “mesofossil” fraction of the Miombo woodland charcoal particle (A) reflected light image (B–D) image processed three-dimensional reconstruction showing all sides of the particle. (E–H) Modern “microfossil” fraction of the Miombo woodland charcoal. (E) reflected light image (F–H) image processed three-dimensional reconstructions showing all sides of the particle.

**Figure 4:**
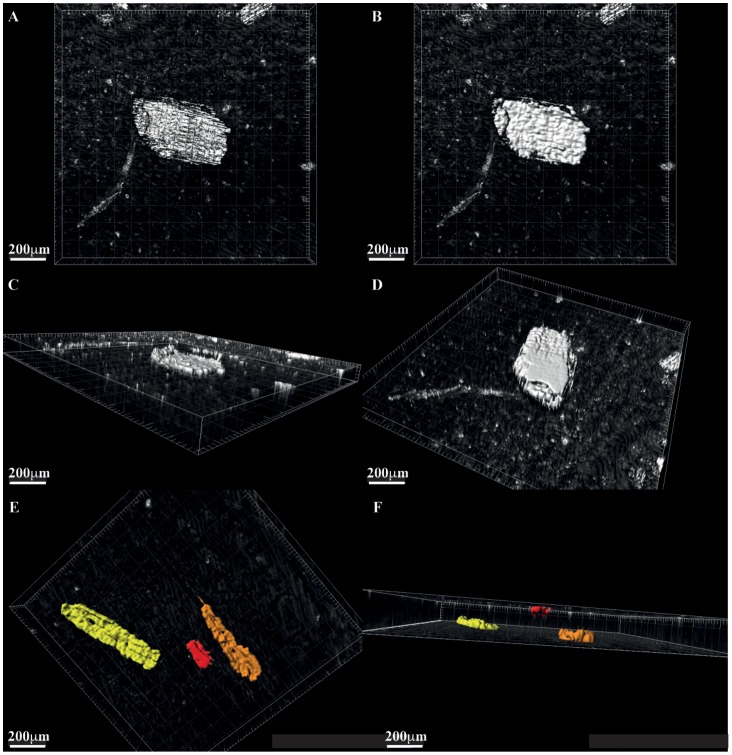
Reconstruction of fossil charcoal particle (A–D) Ancient Cretaceous mesofossil charcoal particle (A) reflected light image (B–D) image processed three-dimensional reconstructions showing all sides of the particle. (E–F) shows also the ability to code the images according to charcoal volume for a range of particles yellow = largest volume red = smallest.

All slides were imaged using a Zeiss LSM710 Inverted Confocal Laser Scanning Microscope (Carl Zeiss, Obercohen, Germany) in the Core Facilities at the Institute of Genomic Biology at the University of Illinois at Urbana-Champaign. Three-dimensional stacked images were collected using Zeiss Zen 2010 software. Because charcoal particles are not fluorescent, they were imaged using reflected light mode from a 633 nm HeNe laser using a T80/20 dichroic mirror where 80% of the incoming laser light is transmitted and 20% reflected. Transmitted light images were simultaneously captured using a transmitted light detector above the condenser. Images were obtained using a Plan Apochromat 10× (0.3 NA) or a 20× (0.8 NA) objective. Image acquisition details are provided in Table 1. The reflection supressing mirror was removed (used in normal fluorescence mode) from the light path, which enabled reflectance imaging. Because the reflection plane or the depth of focus using a given objective is limited by the wavelength and numerical aperture, optical sectioning is possible using this mode. Using the precise (20 nm resolution) automatic z drive attached to the microscope we have taken multiple 2D images through the z axis (axial depth) of the sample. The microscope settings used are shown in Table 1 for the two charcoal size fractions analysed. This microscopy technique allowed the size and shape of particles to be measured in three dimensions from the slides via capture of a set of individual images of each Z-stack focal plane through the particle(s). An example is shown in Figure 1, where the multiple planes of focus are evident from the top to bottom of the particle. The thickness of the slides varied greatly, ranging from 40 µm thick (Miombo microfossil slide) to 215 µm in particle C of the bonfire charcoal. Images were captured concurrently in reflected light (in 3 dimensions, x-y-z) and in bright field illumination (2 dimensions, x–y). We imaged one hundred particles in each slide in an unbiased manner. Imaging of the charcoal particles began at the top left of each slide and each field of view was studied for charcoals following a straight transect from edge to edge of the slide. Every charcoal particle encountered on a transect was imaged until a count of 100 was reached. (This took multiple transects). The reflected light images were processed using the Surpass Module in BITPLANE’s IMARIS 3D volume rendering software (http://www.bitplane.com/go/products/imaris), which is able to piece together the suite of stacked reflected light images e.g. Figure 1 to create a three dimensional reconstruction of the charcoal particle(s)(e.g. Figures 2–4). IMARIS is able to visualise multichannel microscope images in order to provide the optimum information from 2D or 3D images. We used the Surpass View in IMARIS to generate our 3D renderings using the Surfaces visualisation. The user processes each image by thresholding the images to pick up all the relevant voxels (smallest units within the image volume dataset). The thresholding allows all grey values in the image to be assigned a particular functionality (i.e. define the particles of interest). Size limits can be applied in the thresholding so that small objects for example are removed to clear noise (smoothing). Equally large particles can also be screened via this manner (although large particles were not excluded in this dataset). Any irrelevant particles or artifacts that remain in the thresholded images can be removed, using appropriate filters (based on area) and the image reprocessed so that only charcoal particles remain. Once the particles are defined, the Surfaces visualisation module produces computer-generated representations of a specific grey-value range in an image data set. It creates artificial solid objects to visualise the range of interest of a real volume object. The surface is defined by a series of connected triangles where the surface statistics describe the surface mesh (the number of triangles, surface area, enclosed volume). The MeasurementPro module then uses this information to calculate the shape statistics for each particle. This enabled us to estimate the area, volume, shape and sorting of the charred particles in our samples.

**Table 1:** Zeiss LSM710 Laser Scanning Microscope Image Acquisition Settings.

Setting	Large “Bonfire” charcoal and ancient andmodern “mesofossil” fractions	Modern “microfossil” fraction
Image scaling X	2.768 µm	0.526 µm
Image scaling Y	2.768 µm	0.526 µm
Image scaling Z	5.00 µm	1.060 µm
Scan mode	Stack	Stack
Zoom	0.6	1.6
Objective	EC Plan-Neofluor 10X/0.30 M27	Plan-Apochromat 20X/0.82 M27
Pixel dwell	1.58 ms	1.58 ms
Averaging	1	1
Photomultipleor Tube Master gain	Reflection: 551 TRansmission: 359	Reflection: 551 Transmission: 359
Digital gain	1	1
Offset	0	0
Pinhole	38 µm	36 µm
Filters	415–735	415–735
Beam splitters	Master Beam Splitter: T80/R20	Master Beam Splitter: T80/R20
Laser	633 nm: 7.5%	633 nm: 7.5%

## Results

As the first test for this new method we imaged large individual particles of angiosperm charcoal created in a modern bonfire and reconstructed their volume. Figure 2 shows one of the three large particles examined. Included is the captured raw unprocessed reflected light image in the x-y plane, which shows the general x-y shape characteristics of the particle and its surface texture (Figure 2A); the image as processed, with the area mapped out for area and volume estimates in red (Figure 2B), the thickness and axial depth variations of the particle in the Z plane (Figures 2C–D); and the highlighted 3-dimensional properties (Figures 2E–H). Table 2 provides the IMARIS image analysis software generated volume estimates extracted from these reconstructed particles and crude manual estimates for the large “bonfire” particles of charcoal. It can be seen that the manual estimates of the particles volume are significantly larger than those estimated using IMARIS. This is because the manual estimates are based only on the length and width (x-y) of the particles measured from the captured images and the depth of the focal plane through each particle. The manual estimates therefore do not take into account variations in surface topography or the outline of the particles, which can be clearly seen to vary in Figure 2. This comparison highlights the difficulty of measuring the volume of a micro- or mesofossil charcoal particle without computer image analysis software and, therefore, this combined microscopy and image analysis technique does provide a real improvement of our ability to measure such quantities.

**Table 2:** Imaris and Manual estimates of the volume of the large “bonfire” charcoal particles.

Particle	IMARIS	Manual estimate	Manual overestimate
A	0.0132 mm^3^	0.0326 mm^3^	2.47 times
B1	0.0138 mm^3^	0.0385 mm^3^	2.79 times
B2	0.00369 mm^3^	0.0117 mm^3^	3.17 times
C1	0.0460 mm^3^	0.180 mm^3^	3.91 times
C2	0.0361 mm^3^	0.0862 mm^3^	2.39 times

**Figure 5:**
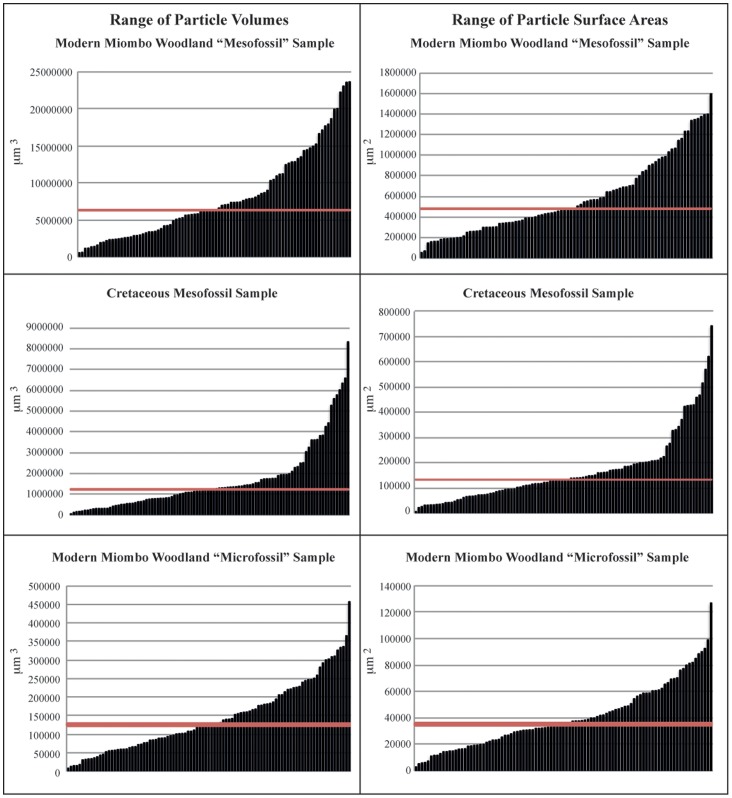
Range of particle volumes and surface areas represented in the samples. Red line is the median for all particles.

Figure 3 shows example three-dimensional reconstructions of micro and meso“fossil” fractions from a modern Miombo woodland wildfire and Figure 4 and video animation Video S1 shows a reconstruction using the ancient Cretaceous mesofossil particles. Using these reconstructions, IMARIS was able to estimate the three-dimensional volume and the two-dimensional surface area of the 100 particles measured in each of our sample types. This allowed us to see the full spectrum of particle volumes and surface areas contained within the samples. Figure 5 shows the full range of measured particle volumes and surface areas ordered in increasing size for all three samples, the red line on the charts shows the median of the measurements. The median particle volume for the samples are 125579 µm^3^, 6313344 µm^3^ and 1227426 µm^3^ for the modern “microfossil”, modern “mesofossil” and the Cretaceous mesofossil samples respectively. The median surface areas are 35225 µm^2^, 478260 µm^2^, 132949 µm^2^, in the same order as listed above (see Table 3 for full size information about the particles). As would be expected, the “microfossil” sample contains considerably smaller particles than either of the “mesofossil” fractions. However, the measurements reveal that the modern “mesofossil” fraction is comprised of much larger particles than the Cretaceous mesofossil fraction. The modern “mesofossil” fraction contains particles that are on average (median) 5 times greater in volume than the Cretaceous mesofossil fraction and the sizes of the particles in the two samples sets can be shown to be significantly different from one another in size (Kruskall Wallis *p* = 1.86×10^−22^). The surface areas of the particles in the modern sample are on average 2 times greater than that of the Cretaceous sample (Kruskall Wallis *p = *1.45×10^−21^). We note that such variations were not obviously apparent to our own eyes during preparation or during confocal laser scanning of the samples. This highlights the utility of this method to determine apparently subtle variations in charcoal size (volume and surface area) between samples.

**Table 3:** Descriptive statistics of the range of sizes of the charcoal particles measured.

	Mean volumeµm^3^	Median volumeµ µm^3^	Standard error	Mean surface area µm^2^	Median surface area µm^2^	Standard error	Minimum particle size	Maximum particle size
Modern Microfossil	143661	125579	9449	40358	35225	2446	7691 µm^3^ 2943 µm^2^	457438 µm^3^ 126843 µm^2^
Modern Mesofossil	8018072	6313344	623915	585030	478260	39020	557041 µm^3^ 53714 µm^2^	23628718 µm^3^ 1596070 µm^2^
Cretaceous Mesofossil	1660692	1227426	161008	167527	132949	13850	25810 µm^3^ 6035 µm^2^	8336841 µm^3^ 742724 µm^2^

**Figure 6:**
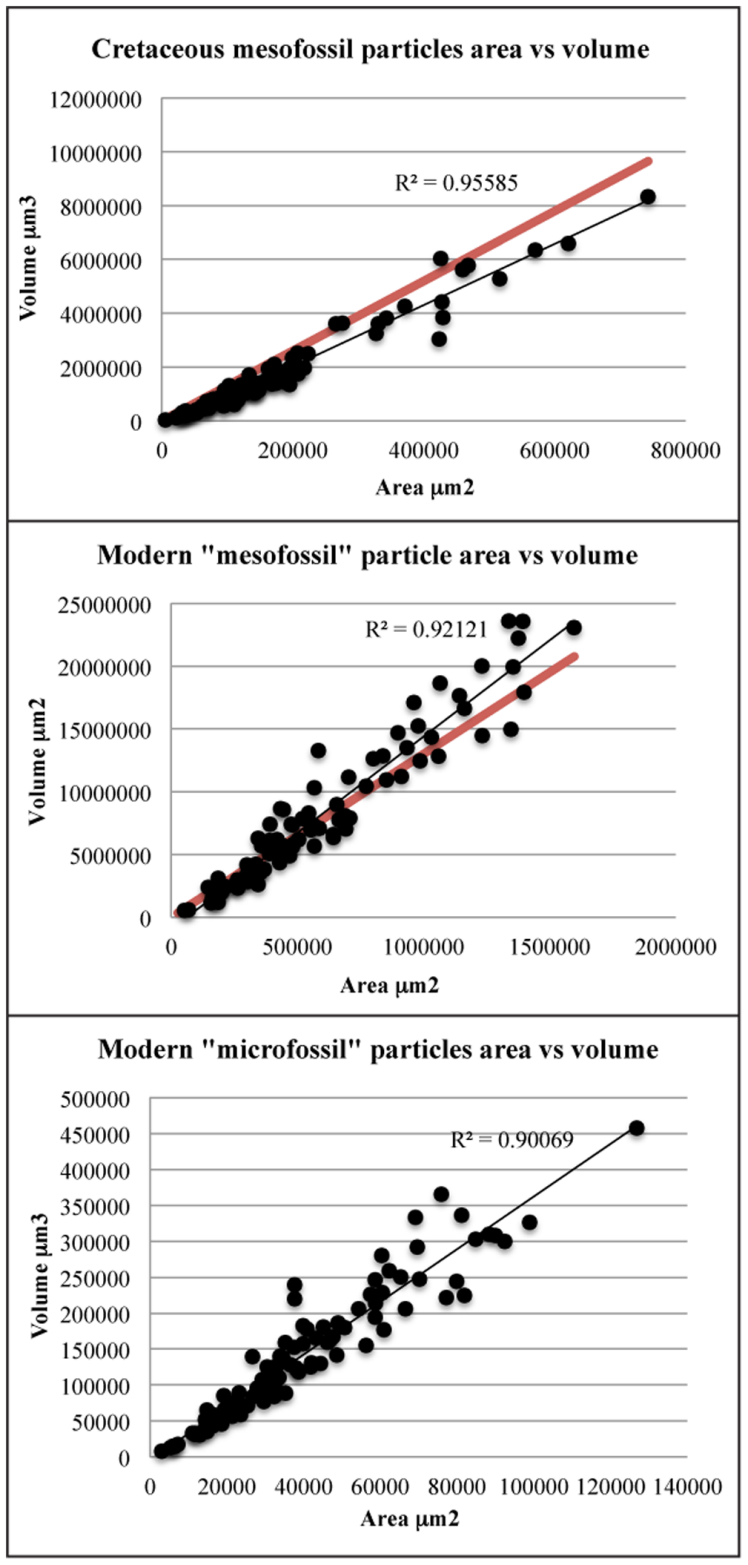
Charcoal particle surface area vs volume relationship for all samples and size fractions. Black line = best fit regression line (with R^2^ shown). Red line corresponds to prediction according to y = 13×.

Figure 6 shows the relationship between measured particle surface area and measured volume for all the samples. In all cases, there was a positive linear correlation (R^2^>0.90 see Figure 6) between the surface area and the volume of the particles. We further measured the “shape” of the particles in the slides using IMARIS, which is able to rate the particles in terms of their relationship to a sphere or an ellipsoid. The majority of the particles in all three samples are characteristically oblate in their nature (i.e. representing flattened spheres or ellipsoids). This explains the good correlation between the surface area and the volume of the particles, as their axial depth (Z) is relatively small compared to their X-Y area. Due to their shallow depth, x-y (areal) measurements will be relatively representative of the total size of the particle because thickness is a small component of the total particle. We might expect that the linear area-volume relationship would break down in larger charcoal particles, which may be more cubic nature (e.g. macrofossil as opposed to mesofossil fractions), and are too thick (high axial depth) to mount reasonably under a cover slip. Or, if the nature of the charred plant material is significantly different, the relationship may not hold; for example, thin charred grass cuticle might have a different linear area-volume relationship to larger fragments of charred wood.

## Discussion

### Impact on Reconstructing Palaeofire Activity

Capturing reflected light images throughout the depth of focus of slide-mounted charcoal particles using a confocal laser scanning microscope has enabled us to create 3-dimensional reconstructions of micro- and mesofossil charcoal particles. This method allows size variations (volume and area) of charcoal particles to be quantified and compared between different samples and accounts for differential fragmentation of particles between samples. This is in contrast to methods which solely estimate total number of charred particles. This provides additional data and a more complete analysis of the nature of particles in a micro- or mesofossil preparation. The samples we have studied reveal that the volume and area of particles can vary greatly between samples and that such changes are difficult to detect with the human eye. As such, when considering only particle number, significant variations in possible fragmentation may be missed and therefore go unaccounted.

As a test for traditional particle count methods, we counted the total number of charcoal particles in the slides imaged for the mesofossil fraction of the Cretaceous and modern samples and estimated the total volume of charcoal in each sample (Table 4) using our three-dimensional rendered volume measurements. When charcoal abundance is compared in the two samples using only the number of charcoal particles, the Cretaceous sample can be shown to contain more particles than the modern sample. If we assumed that our two samples were from a time series of samples from the same location (as would be the norm for a fossil charcoal study) then we might conclude that the Cretaceous sample indicated a period of increased fire activity. However, the estimated total volume of charcoal in the two samples reveals the opposite trend and indicates that in fact the modern meso“fossil” sample contains ∼ 3.4 times more charcoal than the Cretaceous sample. This result indicates that the volume of charcoal particles in a sediment must be estimated in order to build a complete picture of the charcoal assemblage in order to make informed interpretations about past fire histories and that errors in interpretations may result when using only charcoal particle number as a proxy for fire activity.

**Table 4:** Particle count, median particle volume and estimated total volume of charcoal in the sample (calculated by multiplying the median particle volume by the total number of particles in the slide).

Sample	Total Particle Count	Median Volume (mm^3^)	Estimated Total Volume (mm^3^)
Cretaceous mesofossil	216	0.0012	0.2651
Modern mesofossil	142	0.0063	0.8965

### Practical Application to a Suite of Fossil Samples

In our study, we measured a random selection of 100 particles on a single slide from each sample. One hundred particles took approximately 3 hours to acquire as images on the confocal laser scanning microscope and a further 3 hours to create 3-dimensional reconstructions of the 100 particles. The entire Cretaceous mesofossil sample, evenly dispersed over 10 slides, contained ∼3161 charcoal particles. If we had decided to measure the volume of all these particles it would have taken ∼285 hours to image and reconstruct all the particles in the entire sample. It is therefore unrealistic to use this method to measure all the mesofossil charcoal particles from each sample in an entire suite of samples, as would be the normal practice if reconstructing fire activity throughout a sedimentary sequence. The time required to image all particles in a sample could be considered a limitation of the method.

Our suggestion is to use the technique to capture the range of particle volumes represented in a sample by preparing slides of known dispersion and measuring a representative quantity of particles chosen in a non-bias fashion in a slide. We chose to measure 100 particles in our slides, as we felt that this allowed us to capture the typical range of sizes of charcoal represented in our samples. 100 is normally considered sufficient, owing to a sample size of 30 typically being considered large enough for the central limit theorem to take effect. This number also provides a realistic apportionment of time per particle for volume quantitation if acquiring data for a large number of samples in a sedimentary sequence. The measured range of sizes (e.g. Figure 5) can be used to estimate the total volume of particles in the sample by multiplying the mean or median volume by the number of particles in the sample (as counted via traditional methods). Additionally, one could infer approximately how many particles there might be for a range of different size fractions by creating several size groupings from the morphometric data, which would further increase our ability to study differences between the size fractions of charcoal in an assemblage from a time series of samples.

### The Relationship between Particle Surface Area and Volume

We recognise that not all researchers would have access to a confocal laser scanning microscope on a regular basis. The cost of our microscope time was approximately $85 per 100 particles measured, suggesting that in some cases the technique could also be cost prohibitive. In contrast, areal measurements can be taken using a laboratory standard transmitted light microscope with a digital camera attached and with common freeware image processing software (e.g. ImageJ). Therefore, we also attempt to establish relationships between volume and area, and suggest directions where further research is needed to determine whether volume data may be acquired using standard palynology laboratory equipment.

We have shown that there is a positive correlation between the surface area of particles and their volume in these samples. This suggests that (1) measuring particle surface area could provide a useful metric to estimating true variations in charcoal volume between samples and (2) measurements of charcoal surface area might be appropriate for estimating variations in fire activity between samples and throughout time. We suggest that it may also be possible to predict charcoal volume using measured surface area data. As Figure 6 illustrates, charcoal area can be approximately converted to volume by multiplying by a factor of 13. We suggest that the relationship of *y* = 13*x*, where *x* is charcoal area and *y* is charcoal volume, might provide a good general predictive conversion equation to transfer surface area measurements in mesofossil samples to volume estimates.

More samples would need to be analysed, ideally from a range of modern, Quaternary and Pre-Quaternary micro- and mesofossil charcoal preparations, that include charcoals from different plant taxa and organs in order to confirm if such an approach is valid, and in order to generate a globally applicable metric. Measurements of charcoal area would be easily achieved using standard captured images followed by processing in software such as ImageJ making the method more affordable and available to the majority of laboratories. However, even if it were possible under all circumstances to quantify volume based on a particles surface area, we suggest that it may be unrealistic to measure the surface area of all particles in a slide in the case where other black non charcoalified particles exist. This is because such particles are indistinguishable from charcoal using standard image processing techniques that rely on thresholding of images to create precise measurements. Therefore, a similar approach to that used in this manuscript, of measuring 100 particles identifiable as charcoal and multiplying the total number of particles by median surface area, could be used.

Tinner and Hu [13] have suggested that the number of microfossil charcoal particles in Quaternary lacustrine samples could be correlated to the total area of charcoal and, therefore, that the total surface area of charcoal in a sample could be predicted from the abundance of charred particles. We hope that in future work we can further test the relationship between volume and particle number from multiple samples from both Pre-Quaternary and Quaternary samples and from multiple depositional environments. Currently, our charcoal results suggest that this relationship may not be supported for mesofossil fractions because our particle counts do not predict the same volume estimate outcome for the two samples that we compared. This may, however, be due to the difference in preparation techniques of our modern and ancient samples (e.g. our ancient samples were released using acid maceration and the modern were not) and not specific to the geological age of the specimens.

### Concluding Remarks

We have shown that it is possible to create three-dimensional reconstructions of both micro- and mesofossil charcoal particles using a confocal laser scanning microscope coupled to an image analysis system. These reconstructions can be used to generate useful metrics, which provide additional data toward quantifying the abundance of charcoal in Quaternary sediments and Pre-Quaternary rock samples. We have shown that in the case of the samples we studied, our volume estimates reversed the interpretations that might be made if only particle counts had been assumed. This indicates that volume is an important and measurable metric of charcoal assemblages and that it should be considered when interpreting past fire histories.

Our method allows not only the volume and surface area of individual particles to be estimated but allows size sorting within samples to be measured. As such, the method provides considerably more information about the size, shape, volume, range of size classes and total area and total volume of charcoal within a given sample. All these parameters can be used to compare variations between particles throughout a time series of samples. These metrics may provide new information that will enable more information to be gleaned from the fossil record of wildfire events. Areas in which this method could be developed are in interpreting or distinguishing regional versus local fire events, recognition of predominant fire regime (e.g. surface fires are known to favour production of different sized charred particles than crown fires [1]), as well as the possibility to improve our understanding of transport histories (e.g. samples containing a good range of size classes are considered to have been transported shorter distances than those containing relatively few different sizes of particles). A good review of the nature of charcoal production and transport from wildfires can be found in [2].

From visual investigation the majority of charcoals in this study were of charred wood. It is well known that a range of other plant parts are found as fossil charcoals such as flowers, leaves, and plant cuticles (e.g. [1]) A preliminary set of experiments using fragmented oven-charred particles from a range of plant types and organs has shown the potential of the shape metric of circularity to distinguish between charred broad leaves, charred wood and charred fern fronds (CMB, unpublished data). Length-width ratios of charred particles have also been shown to be capable of characterising charred grass remains from that of other leaves [14]. Our 3-dimensional reconstruction method might be well applied to generating and improving upon such morphometric estimates where reconstructions and analyses across a range of charred plant parts are required to test the possibility of image analysis techniques determining proportions of charred wood versus other charred plant parts in fossil residues. This new method, therefore, is likely to be able to provide further useful information to assist us in understanding the nature of past wildfire events and improve our ability to determine the relationship between wildfires and environmental changes in Earth’s past.

## Supporting Information

Video S1 **Movie animation of a 3-dimensional reconstruction of one of the ancient Cretaceous mesofossil particles showing all sides of the particle.**
(MPG)
